# The health impacts of Indonesian peatland fires

**DOI:** 10.1186/s12940-022-00872-w

**Published:** 2022-07-06

**Authors:** Lars Hein, Joseph V. Spadaro, Bart Ostro, Melanie Hammer, Elham Sumarga, Resti Salmayenti, Rizaldi Boer, Hesti Tata, Dwi Atmoko, Juan-Pablo Castañeda

**Affiliations:** 1 Wageningen University and Research, Wageningen, the Netherlands; 2 Spadaro Environmental Research Consultants, Philadelphia, PA USA; 3 University of California, Davis, USA; 4 Dalhousie University, Halifax, N.S. Canada; 5 Washington University in St. Louis, St. Louis, MO USA; 6 School of Life Sciences & Technology, Institut Teknologi Bandung, Bandung, Indonesia; 7 IPB University, Bogor, Indonesia; 8 Center for Climate Risk and Opportunity Management, Bogor Agricultural University, Bogor, Indonesia; 9 National Research and Innovation Agency of Indonesia (BRIN), Jakarta Pusat, Indonesia; 10 Agency for Meteorological Climatological and Geophysics, Badan Meteorologi Klimatologi dan Geofisika (BMKG), Jakarta, Indonesia; 11 Tilburg University School of Economics and Management (TiSEM), Tilburg, The Netherlands

**Keywords:** Health effects, Peatland fires, Indonesia

## Abstract

**Background:**

Indonesian peatlands have been drained for agricultural development for several decades. This development has made a major contribution to economic development. At the same time, peatland drainage is causing significant air pollution resulting from peatland fires. Peatland fires occur every year, even though their extent is much larger in dry (El Niño) years. We examine the health effects of long-term exposure to fine particles (PM_2.5_) from all types of peatland fires (including the burning of above and below ground biomass) in Sumatra and Kalimantan, where most peatland fires in Indonesia take place.

**Methods:**

We derive PM_2.5_ concentrations from satellite imagery calibrated and validated with Indonesian Government data on air pollution, and link increases in these concentrations to peatland fires, as observed in satellite imagery. Subsequently, we apply available epidemiological studies to relate PM_2.5_ exposure to a range of health outcomes. The model utilizes the age distribution and disease prevalence of the impacted population.

**Results:**

We find that PM_2.5_ air pollution from peatland fires, causes, on average, around 33,100 adults and 2900 infants to die prematurely each year from air pollution. In addition, peatland fires cause on average around 4390 additional hospitalizations related to respiratory diseases, 635,000 severe cases of asthma in children, and 8.9 million lost workdays. The majority of these impacts occur in Sumatra because of its much higher population density compared to Kalimantan. A main source of uncertainty is in the Concentration Response Functions (CRFs) that we use, with different CRFs leading to annual premature adult mortality ranging from 19,900 to 64,800 deaths. Currently, the population of both regions is relatively young. With aging of the population over time, vulnerabilities to air pollution and health effects from peatland fires will increase.

**Conclusions:**

Peatland fire health impacts provide a further argument to combat fires in peatlands, and gradually transition to peatland management models that do not require drainage and are therefore not prone to fire risks.

**Supplementary Information:**

The online version contains supplementary material available at 10.1186/s12940-022-00872-w.

## Background

Indonesia faces peat and forest fires every year, with the most severe fires occurring in El Niño years, including 2014, 2015 and 2019 [1]. During these events, smoke extends over large parts of Indonesia as well as Singapore and parts of Malaysia [2]. Much of the smoke is from peatland fires. Out of some 15 million hectares of peat in Indonesia, over half have been cleared and drained, in particular for plantation development (including oil palm and acacia for pulp and paper production) [3]. Once drained, peatlands are susceptible to fires, which can have a significant impact on ambient air quality. Drained, but still wet peat soils burn incompletely, at relatively low temperatures, which results in relatively high emissions of a mix of pollutants including particulate matter, carbon monoxide and Polycyclic Aromatic Compounds (PACs) [2]. The PM_2.5_ (particulate matter with a diameter less than 2.5 μm) concentrations reached in Indonesia during fire events are exceptionally high. For example, in 2015 in Central Kalimantan province, 24-h mean concentrations above 2000 μg/m^3^ were recorded [4, 5], far above short–term exposure levels considered hazardous for human health by the World Health Organization, i.e., 15 μg/m^3^ (24 hour mean) [6].

In the epimidiological literature, it has become common practice to use PM_2.5_ as an indicator of ambient air quality, and causal associations between PM_2.5_ exposure and health effects have been derived. The health effects of particulate air pollution include respiratory and cardiovascular morbidity (e.g., aggravation of asthma, respiratory symptoms and an increase in hospital admissions) and mortality from cardiovascular and respiratory diseases as well as contribute to lung cancer [7]. Given that forest and peatland fires occur across Indonesia each year, and that smoke is retained in the ambient air for several months a year, forest and peatland fires not only lead to spikes in air pollution over the short-term, but also increase the population long-term exposure to PM_2.5_, which is known to have a greater impact on mortality than short-term episodes [8, 9]. Several studies have analyzed health effects of Indonesian peat fires. For example, the short-term health effects of the 1997 fires in Sumatra and Kalimantan were studied in [10] and the long-term health effects in Central Kalimantan province in [11]. Air quality and health impacts of vegetation and peat fires in Equatorial Asia during 2004–2015 were studied in a comprehensive modelling study in [12]. However none of these studies analysed average long-term health effects including both mortality and morbidity effects, based on a comprehensive spatial database of PM_2.5_ concentrations.

The objective of this paper is to analyse the long-term health impact of Indonesian peatland fires over the 5 year period extending between 2013 and 2017. We focus on the lowland provinces of Sumatra and Kalimantan which contain extensive peat areas and where people have the greatest exposures to the fires. We determine (i) the PM_2.5_ ambient air pollution resulting from fires occurring in peat; (ii) exposure of people to PM_2.5_; and (iii) the resulting heath impacts from long-term exposure including adult premature mortality, lost work days, cases of childhood asthma and chronic bronchitis, respiratory hospital admissions and infant mortality. Our paper focusses on Sumatra and Kalimantan where the majority of peatland fires take place and where the health effects of these fires are the highest. We include all types of fire in peatlands including fires affecting above ground vegetation and/or fires that occur in the form of burning and smouldering of below ground peat biomass. Our methodology is novel in the sense that it focusses on peatland fires, and integrates satellite data on air quality, calibrated with data from local monitoring stations, with state-of-the-art epidemiological modelling resulting in a high resolution (4.4 km) model for a large area (~ 1 million km^2^). We also assess the implications of using different concentration-response functions. We acknowledge that health effects of air pollution from peatland fires in Sumatra and Kalimantan occur in a broader area including Singapore and Malaysia (e.g. [13]), but we focus our study on Indonesia, where the fires originate.

## Methods

### Case study area

**Fig. 1:**
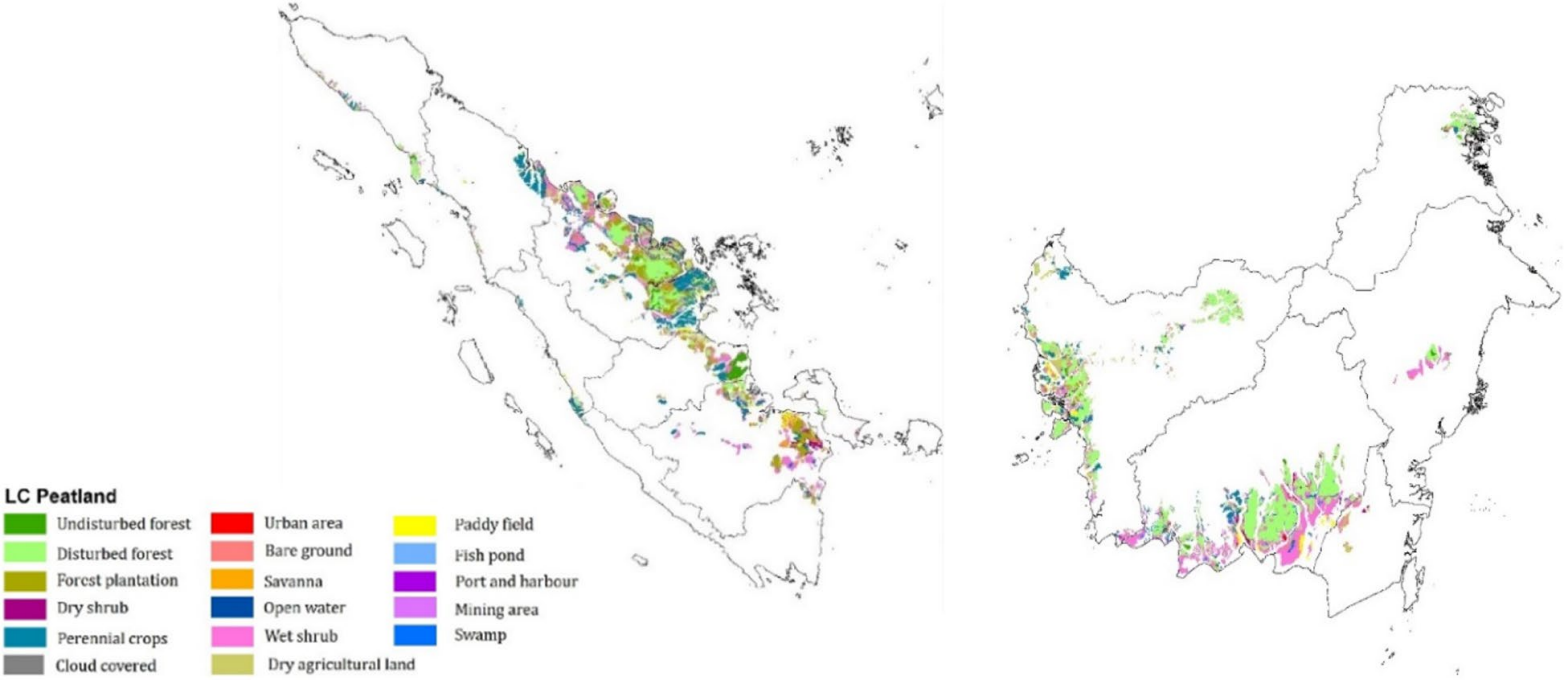
Land cover of peatlands in Sumatra (left) and Kalimantan (right) in 2015. Land cover is from the Ministry of Environment and Forestry [15] and peat extent is from the Ministry of Agriculture [14]

We focus on the health effects of peatland fires in Sumatra and Kalimantan, and while peat and forest fires also occur in Papua and West Papua provinces, their much lower population will result in smaller impacts from the fires. Sumatra covers 48 million hectares (ha). With around 21% of the Indonesian population, it is the most populous island after Java. Sumatra has in total around 6.4 million ha of peat according to government data [14], most of it located along the east coast of the island. Kalimantan is the Indonesian part of the island of Borneo and covers around 54 million ha. Based on government data of the Ministry of Agriculture [14], Kalimantan has approximately 4.9 million ha of peatland. Peat occurs in all provinces of Kalimantan: South, Central, West, East and North Kalimantan. Large scale deforestation and peat conversion started in the 1980s, initially in Sumatra. In recent years it has slowed down in response to government policies, but peat conversion is still ongoing in particular for the cultivation of oil palm. The peat extent, and land cover in the peatlands of Sumatra and Kalimantan are shown in Fig. 1. Peatland fires typically occur in the dry season – starting in June and extending to December in some parts of Indonesia – with most fires taking place in the period from August to October.

### Peatland fire occurrence

The burned areas in Sumatra and Kalimantan in each year from 2013 to 2017 were derived from satellite imagery collected through MODIS (MCD64A1 Collection 6). The MODIS MCD64A1 product uses a burned area mapping algorithm to detect changes in vegetation using the optical MODIS sensors [16], providing daily data with a 500 m grid resolution. Daily MODIS MCD64A1 data were downloaded as shapefiles, aggregated by year, and overlaid with the Government of Indonesia Ministry of Agriculture peat map from 2011 [14], in order to identify which fires took place in areas that have peat soils (as opposed to mineral soils). The number of fires varied considerably between years, with over 100,000 individual fires recorded during the particularly dry year 2015.

In order to specify the type of land cover where the burned areas were identified, the results were subsequently overlaid with the land cover maps of the Ministry of Environment and Forestry from 2013 to 2017. These land cover maps show the spatial extent of 22 classes of land cover, which are aggregated here into five broad categories: 1) forests (intact and degraded peat swamp forest); 2) plantation (including oil palm, hevea rubber and coconut) and forest plantation; 3) degraded lands (bare ground, wet/dry shrubs, savannas, and grassy areas); 4) agricultural lands; and 5) other categories. For each of these classes, emission factors were calculated, in order to estimate which proportion of ambient PM_2.5_ is derived from fires in peatland (discussed below). All spatial analyses were conducted in the ArcMap 10.5 GIS package.

### Ambient PM_2.5_ from peatland fires

#### Estimating ambient PM_2.5_ concentrations from satellites and simulation

Due to the limited availability of ground measurements of PM_2.5_ in Indonesia, we use estimates of surface PM_2.5_ concentrations derived from a combination of satellite remote sensing and chemical transport model simulation [17]. Monthly estimates of surface PM_2.5_ concentrations are derived over Sumatra and Kalimantan for 2013 to 2017 at a spatial grid resolution of 4.4 km. PM_2.5_ concentrations are estimated using satellite-derived Aerosol Optical Depth (AOD). AOD is a measure of the extinction (through scattering and absorption) of light by aerosols in a column of air from the Earth’s surface up to space. The AOD is used to represent the abundance of total aerosol particles in an atmospheric column [18]. The daily surface PM_2.5_ concentrations from each data source are obtained by applying the daily simulated AOD to PM_2.5_ ratios to the coincident daily calibrated AOD sources (see Supplementary Materials). Monthly means are calculated from the daily PM_2.5_ values. The monthly mean PM_2.5_ concentrations from each source are then combined based on their relative uncertainties with AERONET following [17]. Details are presented in the Supplementary Materials. Modelled PM_2.5_ concentrations, from AOD, are calibrated with PM_2.5_ and PM_10_ data from Indonesia’s air quality monitoring network, that comprises 16 stations in Sumatra and 13 stations in Kalimantan, providing daily data from 2015 to 2017 (operated by the Meteorological, Climatological and Geophysical Agency and the Ministry of Environment and Forestry) [19, 20]. The majority (90%) of PM emitted from peatland fires is in the fine particulate fraction (PM_2.5_) [2].

#### Identifying the contribution of peatland fires to ambient PM_2.5_

The MODIS data show that fires almost exclusively take place in the period June to December. The Indonesian dry season varies somewhat between the southern part of Kalimantan and the northern part of Sumatra, but generally lasts from May to October. Hence, the peat burns in the dry season as well as in the beginning of the wet season, especially in El Nino years such as 2015 when major parts of Sumatra and Kalimantan were unusually dry from September to November [21]. Consequently, in Sumatra and Kalimantan, the months with high PM_2.5_ concentration in the peat areas (above 50 μg/m^3^) are all during the period June to December, with the highest exposures occurring during the months from August to October (August being the peak of the dry season). This is contrary to Java, which is much more industrialized and more densely populated, and where concentrations exceeding 50 μg/m^3^ occur throughout the year. Note that, due to prevailing wind directions during the dry season, the peatland fire smoke generally does not reach Java [22]. Hence, we assume that the increment in the PM_2.5_ concentration during the dry season, that we observe throughout Kalimantan and Sumatra, can be attributed to fires. To make sure all dry season peatland fires are included in the analysis, based on the rainfall distribution over the years of the analysis, the peat fire season is defined, for the purpose of this analysis, as the period between June and December. The period January to May is defined as the wet season, where we assume there are no peat fires. For each province, the mean concentration during the peat fire season and during the wet season is calculated. The difference between these two is assumed to represent the contribution of biomass fires to ambient PM_2.5_. This difference is then multiplied by 7/12 to calculate the annual average increase in PM_2.5_ concentration, which we use to quantify the health impact of peatland fires. This is because we relate health effects to the mean annual increase in PM_2.5_ as a consequence of peatland fires, in line with the epidemiological evidence that the health effects of long-term exposure are much more important than the impacts from peak exposure [8, 9]. The outcome of the analysis is not sensitive to the assumed length of the peat fire season. If it is assumed that the peat fire season is 6 months, for example, a higher monthly average increase during this season would be noticed, but this would be multiplied with 6/12, resulting in approximately the same annual increase in ambient PM_2.5_ due to peatland fires.

Next, we analyze which part of the incremental PM_2.5_ can be attributed to fires in peatlands, as opposed to PM_2.5_ originating from fires occurring in ecosystems on mineral soil (for instance from forest fires or the burning of biomass in crop fields). For each island, we assume that the increase in the concentration of PM_2.5_ from peatland fires is proportional to the share of PM_2.5_ emitted in peatland fires as opposed to PM_2.5_ emitted from fires on mineral soils. We estimate this proportion based on the number of hectares burned in peat versus mineral soil, taking into consideration differences between years, type of ecosystem and emission factors (EF). We acknowledge that the formation of secondary organic aerosols (SOA) may differ between peatland and forest fires, but we have not taken this difference into consideration in the analysis. We comment on the consequences of our simplification in the Discussion section. The burned areas are analyzed with MODIS images. The location of the fires is compared to the land cover map of MOEF [23], and the national peat map [14]. The amount of biomass burned (above ground biomass and, in peat soil, soil biomass) per hectare was analysed for various ecosystem types (forests, plantations, shrubland, grassland and cropland). It was assumed that on average peat soils burn to a depth of 33 cm, and that per hectare of burned peat 505 t of underground biomass is lost [23–25]. The resulting EF for peat is 4.6 t PM_2.5_ per hectare burned [26]. For above ground biomass, the EF ranges from 0.03 t PM_2.5_ per hectare for fires in agricultural land to 3.5 t PM_2.5_ per hectare for fires in forests [27, 28]). It is assumed that these numbers apply to both ecosystems on peat and on mineral soil. For peatland we add the PM_2.5_ emissions from peat burning (below ground) to the emissions from above ground biomass burning to calcuate the overall EF. Supplementary Materials (SM) Table A2 in the Annex provides detailed calculations.

Finally, for each province, month and year the population weighted increase in PM_2.5_ concentration due to peatland fires was calculated. This is important since the populations are not homogeneously distributed across provinces, and, consequently, the spatially average concentrations do not correlate perfectly with the true population exposure. We used population data from the NASA Socioeconomic Data and Applications Center (SEDAC), file: Gridded Population of the World 2015.^1^ This dataset contains population data at 1 km grid cell resolution, world-wide. In our analysis, for every grid cell, the increase in PM_2.5_ concentration in the grid cell is multiplied with the population in the grid cell. Next, these numbers are added for all grid cells in a province, and the resulting number is divided by the number of people in the province. This results in the population-weighted average increase in PM_2.5_ concentration due to peatland fires by province.

### Health effects from peatland fires

We follow a health impact assessment approach to calculate the health effects of peatland fires from long-term exposure (1-year or longer) to PM_2.5_ ambient air concentrations. We did not specifically calculate the health impacts attributable to episodic events of air pollution, although these short-term effects are implicitly included in the epidemiological assessment of the average long-term health consequences. When analyzing the impact of PM_2.5_ on human health, four main inputs are used to quantify effects on people: (1) current and counterfactual PM_2.5_ concentrations to determine the change in PM_2.5_; (2) size and age-composition of population groups exposed to current levels of air pollution; (3) baseline incidence of mortality and morbidity outcomes; and (4) risk functions relating a change in concentration to a change in the health outcome of interest. We analyze the annual average health effects of peatland fires, relating annual average health effects to annual average PM_2.5_ concentrations (in line with, e.g., [8, 9, 29, 30]. In reality, variation between years may occur since peak concentrations in PM_2.5_ that exacerbate health effects in the short term occur more frequently in dry years with more peatland fires. To capture the variability of the impact results, we also conduct a sensitivity analysis to the key input factors in the analysis, as described below. The health impact is calculated as follows:

$$Health\kern0.5em Impact= Exposed\kern0.5em population\kern0.5em \times \kern0.5em Background\kern0.5em rate\kern0.5em of\kern0.5em mortality\kern0.5em or\kern0.5em morbility\kern0.5em \times \kern0.5em Health\kern0.5em risk\kern0.5em function,\kern0.5em CRF\kern0.5em \times \kern0.5em Change\kern0.5em in\kern0.5em pollution$$

#### Current and counterfactual PM_2.5_ concentrations

The health impacts of air pollution related to peatland fires are compared to the health effects of air pollution that would occur without peatland fires (in which case there would still be a certain level of air pollution, e.g. from industry or traffic). This background air pollution concentration is referred to as the “counterfactual”, the ambient air concentration that would be expected in the absence of peatland fires, and the dry season excess exposure above the counterfactual is used to evaluate the excess health impact attributed to peatland fires. In the assessment of the long-term adult mortality, the incremental PM_2.5_ from peatland fires is defined as the 5-year average of the population-weighted, province-specific concentrations from June to December (fire season) minus the concentrations from January to May (the counterfactual). On the other hand, infant mortality and morbidity impacts are calculated based on the mean PM_2.5_ incremental exposure above the counterfactual in a given year.

#### Size and age composition of population groups exposed to current levels of PM_2.5_

For each province in Sumatra and Kalimantan, annual population statistics, including size and age-group stratification, were calculated according to the Indonesian Statistics Agency and Bappenas population scenario [31]. In 2013, the population of Sumatra and Kalimantan was 53.5 and 15.0 million persons, with an average annual population growth rate between 2013 and 2017 equal to 1.2 and 1.5% for each region, respectively. The average population composition over the 5-year period by broad age-group including infants, children (ages 1 to 14 years), young adults (15 to 30 years) and adults older than 30 years was 2.2, 27.7, 26.2, and 43.9%. Age structure was similar for both islands.

#### Baseline rates of mortality

Next, we estimate the underlying rate of the health effect (e.g., the baseline natural mortality rate, excluding accidental deaths, in the population in terms of deaths per thousand people). We found two sources for these data, so a sensitivity analysis was conducted. For the base case, the data used were taken from the Global Health Estimates (GHE) database by WHO [32], which provides all-cause and cause-specific mortality estimates based on official Indonesian national statistics as of year 2016 (the last year available for such data). For year 2016, the GHE indicates 12.56 natural deaths per 1000 adults aged 30 and older, and 9.53 deaths for non-communicable diseases and lower respiratory illness combined (NCD + LRI) per 1000 adults 25 and older in Indonesia. These baseline mortality rates have been applied, without further adjustment, to population estimates from the Bappenas population scenario for each of the 5 years between 2013 and 2017 to calculate the annual baseline mortality [31]. For infant (< 1 year) mortality, modeled data were obtained from the United Nations Inter-agency Group for Child Mortality Estimation [33]. The annual infant mortality rate in Indonesia over the period 2013 to 2017 was: 25.25, 24.34, 23.46, 22.62, and 21.86 deaths per 1000 live births. These data were used in the calculation of the annual infant mortality.

For the sensitivity calculations, we used modeled demographic data from the Global Health Data exchange (GHDx) database of the Institute for Health Metrics and Evaluation.^2^ At the national-level, IHME estimates for year 2016 are 11.09 natural deaths per 1000 adults over 30 years, and 8.42 NCD + LRI deaths per 1000 adults aged 25 and over. Infant mortality figures are fairly close to the values by the UN IGME [33]. The IHME database also provides modeled mortality rates by province (e.g., Aceh, West Kalimantan, etc.). For Sumatra and Kalimantan islands, respectively, the mortality rates are 5.50 and 6.41 natural deaths per 1000 population (compared to 6.71 per 1000 at the national-level from WHO’s GHE database), 10.05 and 11.40 natural deaths per 1000 adults over 30 years, and 7.56 and 8.49 NCD + LRI deaths per 1000 adults 25 and over.

#### Concentration-response functions

The fourth input is based on statistical relationships from the epidemiological literature that relate ambient concentrations of PM_2.5_ to mortality and other health effects. Over the past decades, epidemiological studies on biomass-derived PM have investigated the mortality and morbidity effects of wildfires on respiratory and cardiovascular outcomes [34–36];;. While the toxicological evidence is suggestive that wildfire PM is more toxic than urban PM, we have adopted the conservative assumption that all particles are equitoxic, and have applied in our analysis the most robust CRFs recently derived on the basis of all PM sources. The accuracy of the risk functions depends in part on: (a) the data quality and methodology of the original studies; (b) the extent to which the concentration-response functions (CRF) include the range of concentrations for which they are being applied, and (c) the applicability of these functions to populations besides those from which they were originally estimated.

For the base-case health assessment of adult mortality from exposure to ambient PM_2.5_ resulting from peatland fires, a meta-analysis was used [30]. This study included 53 cohort studies, 39 studies from North America, eight from Europe, and six from Asia. The mean concentration of PM_2.5_ across the studies was 15.7 μg/m^3^, with higher concentrations observed in the Asian studies with a mean of 30.5 μg/m^3^. The authors’ analysis indicated robust association between PM_2.5_ and premature mortality. At the mean concentration of 15.7 μg/m^3^, a 10 μg/m^3^ increase in PM_2.5_ was associated with a 10.3% (95% confidence interval (CI) = 9.7, 11.1%) increase in adult premature mortality. This risk function was used for the base case estimates since it included the greatest number of studies in total, with a few from Asia. The referenced health risk was applied to the mortality rate in the population over 30 years old [30].

A sensitivity analysis was performed by using three alternative concentration-response functions. The first is an estimate of adult mortality; a meta-analysis of 41 cohort studies from around the world that was conducted while allowing for flexibility in determining the shape of the CRF [9]. The second study [37], utilized census data on 2.4 million Canadian adults to examine non-accidental and cause-specific mortality between 2001 and 2011. Of particular interest, the study compared impacts of using three different average exposure periods (1, 3, and 8 years), so it is relevant to the five-year average of concentrations due to peatland fires. The health risk from [37] was applied to the mortality rate in the population over 30 years old. The third study used in the sensitivity analysis is based on the recommendations from WHO’s HRAPIE revie w[7], which was based primarily on an earlier meta-analysis [8]. The estimated risk for non-accidental mortality (for the population 30+) was based on eleven studies available at the time. The resulting risk was 6.2% (95% CI = 4, 8.2%) per 10 μg/m^3^ increment in PM_2.5_ concentration. As shown in Annex 2, the CRF that we use in our base case [30], lies in between those of the three other studies.

Infant mortality (age < 1 year) was assessed based on [38]. This study considered almost 1 million births across sub-Saharan Africa using the Demographic and Health Surveys, a set of nationally representative samples from 30 sub-Saharan countries. PM_2.5_ was estimated at the residential location using remote sensing satellite data and assigned to each birth using exposures 9 months before and 12 months after birth. The study reported that a 10 μg/m^3^ change in PM_2.5_ was associated with 9.2% (95% CI = 4, 14%) increase in infant mortality. Previous studies in Mexico City [39, 40] generated risk estimates of 8.8 and 6.9%, respectively, for a 10 μg/m^3^ change in PM_2.5_. Finally, another large meta-analysis [41], included over a half million births from 69 nationally representative Demographic and Health Surveys conducted in 43 countries throughout Africa, the Middle East, Asia, Eastern Europe and the Caribbean. The study suggests an increase in infant mortality of 8.1% (95% CI = 2.5, 14.3%) for a 10 μg/m^3^ change in fire-related PM_2.5_, which is quite similar to the result of [38].

For morbidity, based on available data, the following were quantified: severe asthma attacks in children, asthma-related hospital admissions (all ages), all respiratory hospital admissions (all ages), and lost work days for the current labor force. Thus, outcomes such as cardiovascular morbidity (heart attacks, hospital admissions), respiratory symptoms leading to restricted activity and adverse birth outcomes (besides infant mortality) were not quantified because of uncertainties regarding baseline levels in Sumatra and Kalimantan. Most of the CRFs were based on WHO’s HRAPIE [7], except one study [42], which was used for asthma-related hospital admissions. Baseline prevalence rates were determined as follows: severe child asthma attacks were assumed to be 1.24 cases/year per child, assuming 62 asthma attacks a year, of which 2% are severe ([7, 43];, respiratory hospital admissions were assumed as 397 per 100,000 people [44],and lost work days were 7 days/worker (UN ILO). For asthma-related hospital admissions, we were unable to find baseline evidence for Indonesia, instead, we used evidence from Europe, and assumed that 11% of all respiratory hospital admissions are asthma-related.

## Results

### Peatland fire occurrence

Between 2013 and 2017, peatland fires occurred every year in Sumatra and Kalimantan (Table 1), with the largest burned areas detected in 2014 (0.72 million ha) and 2015 (0.42 million ha). The three provinces with the largest burned areas were: Riau, Central Kalimantan and South Sumatra. On average, over the period 2013–2017, 2.3% of all peatlands in Sumatra and 3.0% of all peatlands in Kalimantan burned each year. However, there were large variations between years. For instance, in 2014, 5.4% of all peatlands burned in Sumatra, and 7.8% in Kalimantan. Within the peatlands, most fires occurred in degraded lands (bare ground, wet and dry shrub, savanna and grassland), totaling 52 to 80% of total burned areas annually, followed by plantations (6 to 37%), agricultural lands (3 to 13%) and forests (including primary and degraded forest) (4 to 10%; see Annex 1, Table A1, for details). The largest fires in degraded lands occurred in 2014 and 2015 when 0.46 million ha and 0.34 million ha of degraded peatlands were burned, respectively.

**Table 1:** Peatland fires in Sumatra and Kalimantan

Fire occurrence	2012	2013	2014	2015	2016	2017
**Sumatra total (1000 ha)**	**364**	**274**	**643**	**630**	**96**	**23**
- of which: peatland fires	39%	64%	53%	28%	50%	28%
- of which: fires on mineral land	61%	36%	47%	72%	50%	72%
**Kalimantan total (1000 ha)**	**342**	**105**	**984**	**1008**	**46**	**25**
- of which: peatland fires	55%	45%	39%	24%	16%	21%
- of which: fires on mineral land	45%	55%	61%	76%	84%	79%

### Ambient PM_2.5_ from peatland fires

The monthly satellite-derived (SAT) surface PM_2.5_ concentration estimates for Indonesia are compared against monthly ground monitor data (Insitu) in Table 2. The comparison is shown for ‘All Sites’ (direct PM_2.5_ sites and the sites where PM_2.5_ is calculated from PM_10_), and for direct PM_2.5_ sites (Direct PM_2.5_ Sites). The ground measurements only cover the period of 2014 to 2017. The majority of the ground-based observations over Indonesia provided PM_10_ concentrations rather than PM_2.5_. The PM_10_ measurements were used to estimate PM_2.5_, based on sites where there were coincident PM_2.5_ and PM_10_ values. The column ‘All Sites’ in Table 2 contains the analysis for all sites (the sites measuring directly PM_2.5_ values as well as the sites where PM_2.5_ was estimated from PM_10_), while the column ‘Direct PM_2.5_ Sites’ is referring to the analysis for only sites which directly measured PM_2.5_.

**Table 2:** Comparison of monthly collocated satellite-derived PM_2.5_ (SAT PM_2.5_) and ground monitor PM_2.5_ (Insitu PM_2.5_) over Indonesia for 2014 to 2017)

	All Sites	Direct PM_2.5_ Sites
R^2^	0.77	0.90
Slope, intercept	0.78, 11.29	1.07, 6.90
Mean SAT PM_2.5_ (μg/m^3^)	33.81	33.53
Mean Insitu PM_2.5_ (μg/m^3^)	28.39	24.39
No. of observations	364	71

There is a high degree of consistency (*R*^2^ = 0.77 for all sites; *R*^2^ = 0.90 for direct PM_2.5_ sites) between the PM_2.5_ estimates and the independent ground monitor data, with slopes of 0.78 for all sites and 1.07 for direct PM_2.5_ sites and no significant differences in performance between Kalimantan and Sumatra. The mean satellite-derived PM_2.5_ over the entire 2014–2017 period (33.81 μg/m^3^ for all sites; 33.53 μg/m^3^ for direct PM_2.5_ sites) demonstrate a good agreement with the mean ground monitor PM_2.5_ (28.39 μg/m^3^ for all sites; 24.39 μg/m^3^ for direct PM_2.5_ sites). This comparison with ground monitor data indicates that the satellite-derived PM_2.5_ estimates are accurately capturing the high PM_2.5_ concentrations associated with biomass burning events, and can be used with a high degree of confidence for estimating the impacts of PM_2.5_ due to biomass burning over Indonesia.

**Fig. 2:**
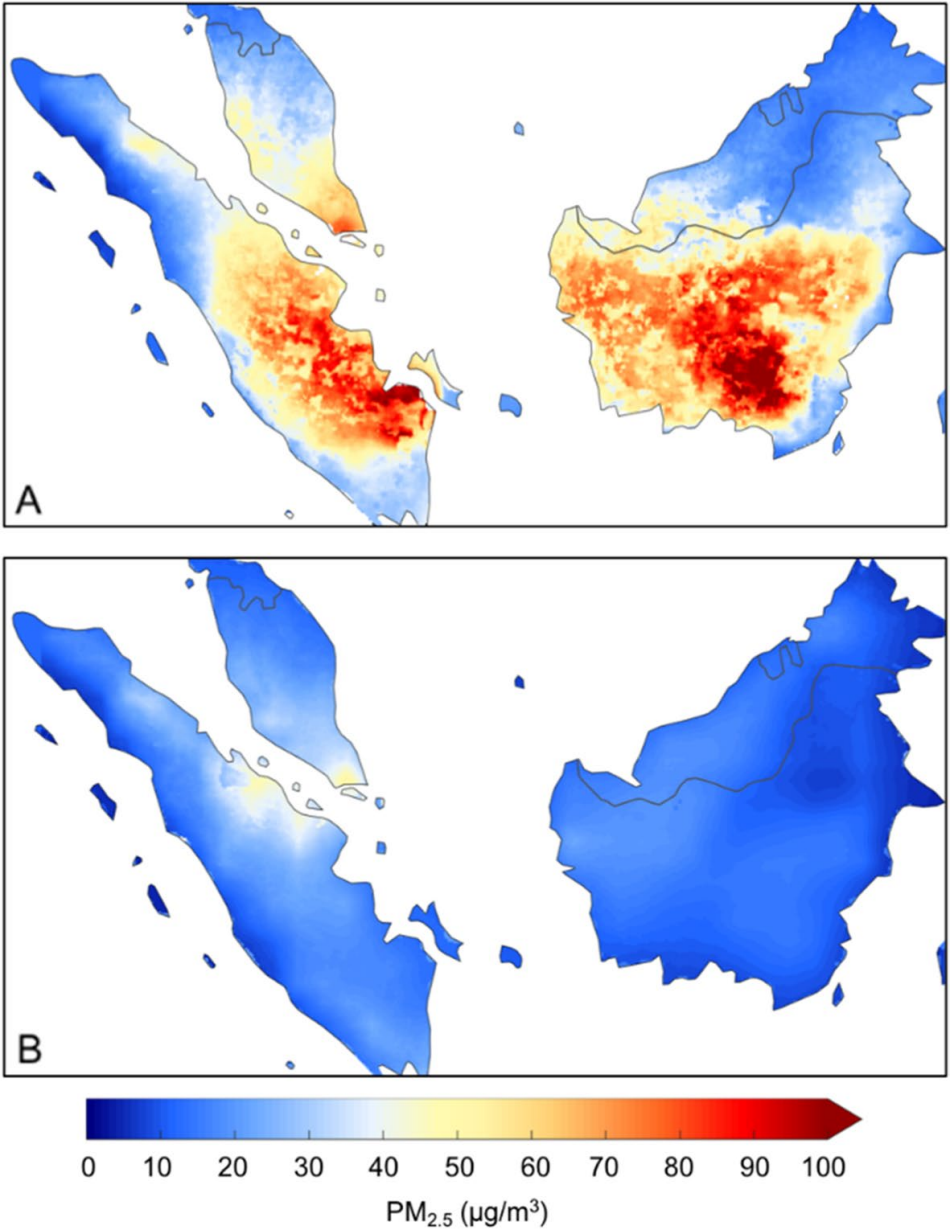
The 2013–2017 average PM_2.5_ concentrations for (**A**) August-Oct (core fire season) and (**B**) all other months for Sumatra and Kalimantan

Figure 2 (A) showsthe 2013–2017 average PM_2.5_ concentrations for the core fire season (months of August to October) and (B) all other months in Sumatra and Kalimantan. As shown in Fig. 2 (A), hotspots are apparent predominantly in the fire season, in particular in the southeastern part of Sumatra (Riau, Jambi and South Sumatra provinces) and in the provinces of Central and West Kalimantan

**Fig. 3:**
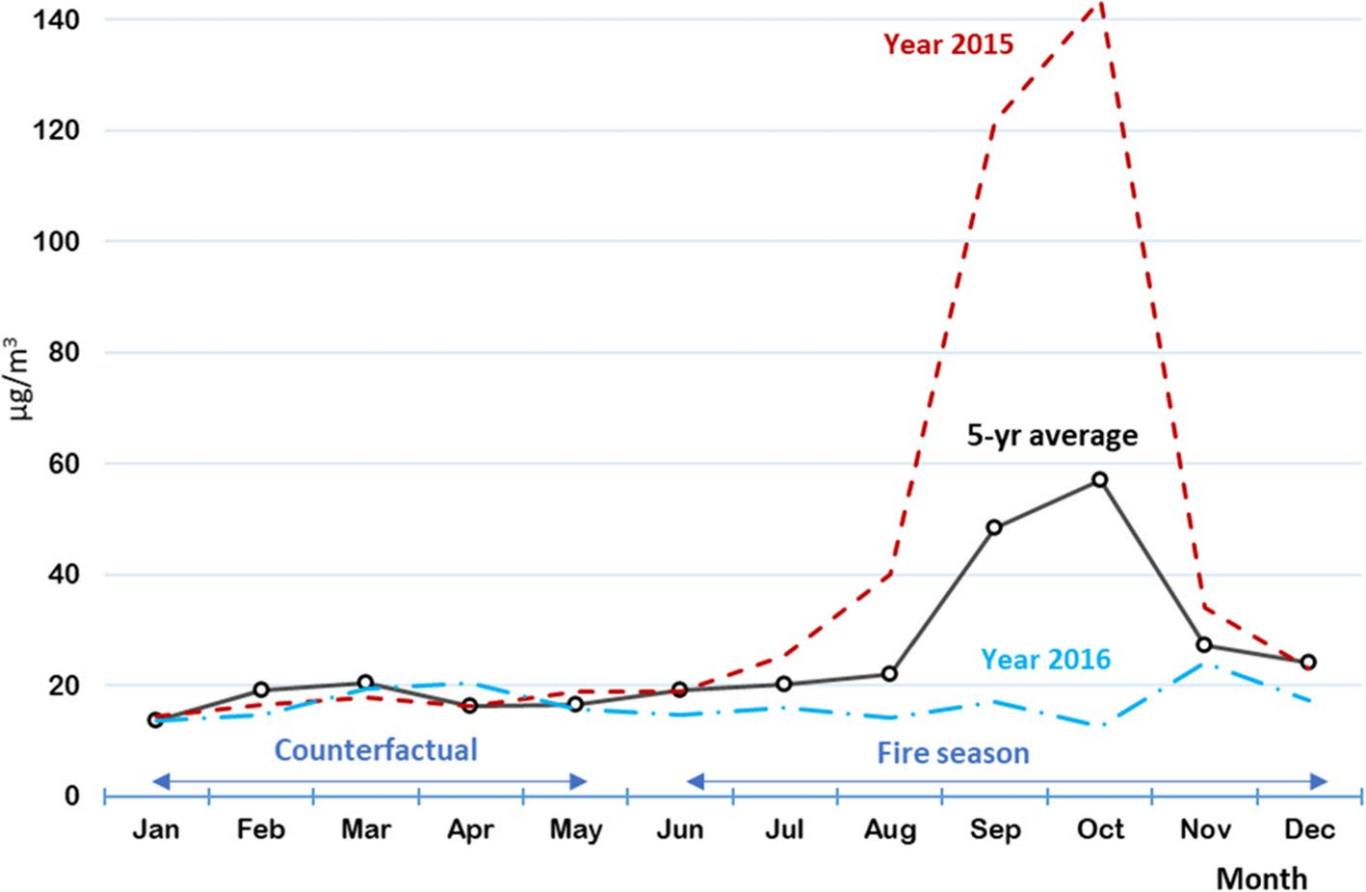
Monthly averaged population-weighted PM_2.5_ concentrations (μg/m^3^) over a five-year period from 2013 to 2017 across the islands of Sumatra and Kalimantan

Figure 3 shows how PM_2.5_ concentrations vary across the year, indicating both the 5-year average and the concentrations for a dry (2015) and wet (2016) year. During the fire season (June to December), average PM_2.5_ concentrations in Sumatra and Kalimantan reach almost 60 μg/m^3^ (with a maximum monthly average PM_2.5_ concentration of 143 μg/m^3^ in October 2015), while for the non-fire season months, average concentrations are around 20 μg/m^3^. Note that these maps show PM concentrations as determined by all emission sources including fires on peat and mineral land, industry, traffic, etc. The difference between the average PM_2.5_ concentration during the 5 months wet season (the ‘counterfactual’) and the 7 months dry season is assumed to be the contribution of biomass burning comprising both fires in areas with a mineral soil and in areas with a peat soil.

Supplementary Materials Table A5 presents the contribution of peatland fires to the overall PM_2.5_ emission from biomass burning. On average, over 5 years, this contribution is 76% in Kalimantan and 86% in Sumatra. Subsequently, the population-weighted exposure to PM_2.5_ from peatland fires by province and by year are calculated. This is shown in Table 3. The table shows the average PM_2.5_ exposure of people living in each province.

**Table 3:** Mean annual increase in PM_2.5_ concentration (μg/m^3^) due to peatland fires, by year and province

Province	2013 (typical rainfall)	2014 (dry year)	2015 (dry year)	2016 (wet year)	2017 (wet year)	2013–17
Sumatra	5.4	7.8	16.8	0.9	2.2	6.6
Aceh	1.2	0.0	3.2	0.0	0.8	1.0
Jambi	10.4	19.5	34.5	1.9	4.3	14.0
Riau	12.5	20.5	26.1	0.0	1.2	11.9
South Sumatra	10.8	19.0	33.4	3.5	5.1	14.3
North Sumatra	0.8	0.0	8.3	0.0	0.0	1.8
Bangka Belitung	6.5	8.4	17.4	4.6	3.4	8.0
Riau Islands	7.6	7.3	13.3	4.1	1.9	6.8
Lampung	5.8	4.2	8.5	0.0	4.3	4.5
West Sumatra	0.0	1.8	18.5	0.0	0.6	4.2
Bengkulu	5.6	7.1	16.5	0.0	3.0	6.4
Kalimantan	4.1	13.3	21.3	0.6	0.9	8.0
West Kalimantan	4.6	12.9	22.8	0.6	0.5	8.2
South Kalimantan	3.2	10.6	18.2	0.7	1.4	6.8
Central Kalimantan	4.2	25.1	33.1	0.0	1.2	12.6
East + North Kalimantan	4.1	9.4	15.3	0.8	0.9	6.0

### Health effects from peatland fires

**Fig. 4:**
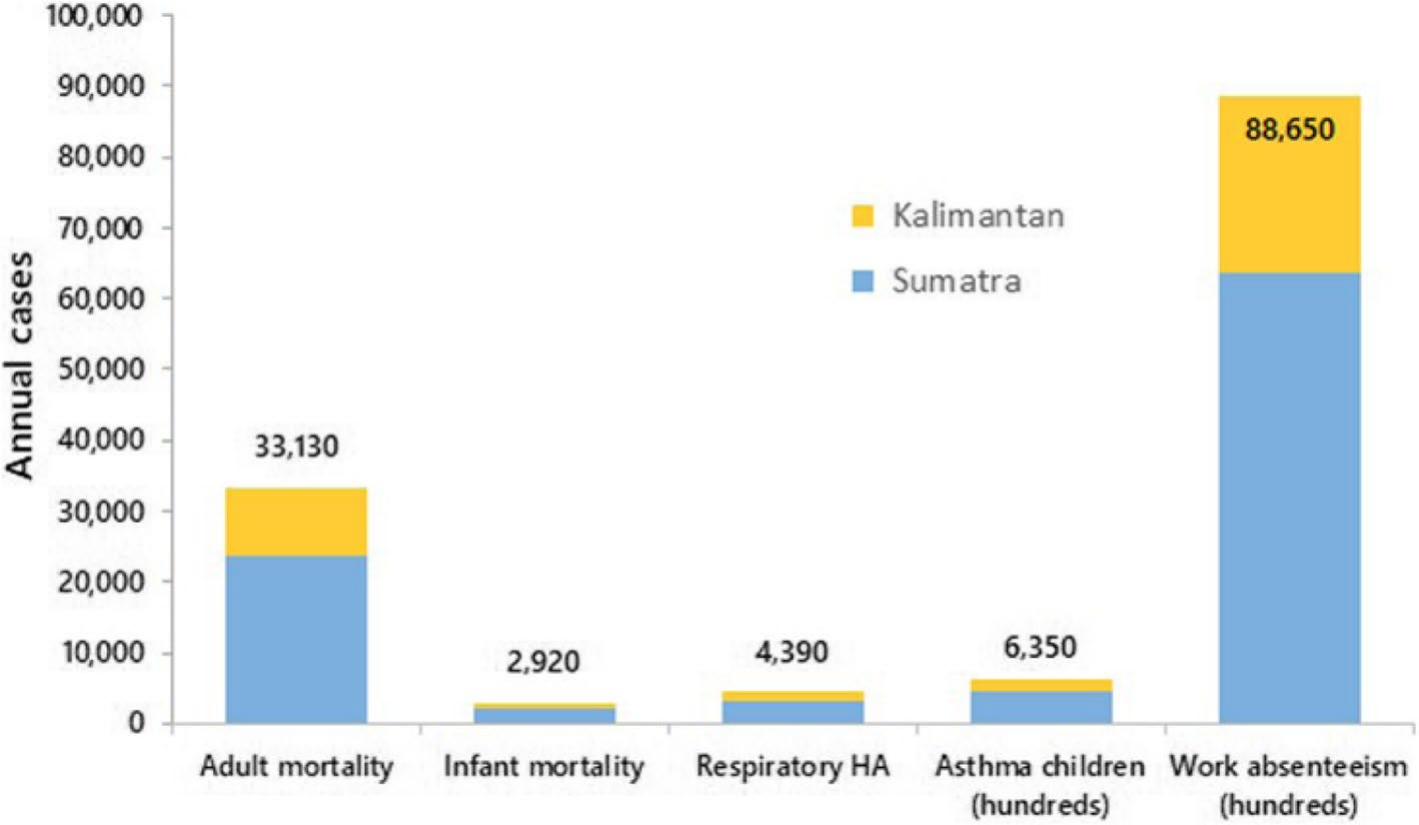
Health impacts of Indonesian peatland fires, based on a CRF curve of [30]

Health impacts of fires in Indonesian peatlands, in Sumatra and Kalimantan, are shown in Fig. 4. Using the CRF curve of [30], each year around 33,100 adults (95% CI = 31,200, 35,700) and around 2900 infants die prematurely because of air pollution resulting from peatland fires. This mortality rate corresponds to around 10% of total adult mortality (over 30 years old) in Sumatra and Kalimantan and 7.7% of the annual infant mortality. For a different perspective, the loss of life expectancy due to peatland fires at birth per inhabitant in Sumatra and Kalimantan is, respectively, 0.9 and 1.2 years (life expectancy at birth is around 71 years). These estimates were calculated using a life table analysis [45], considering province-specific data on the population and mortality distribution by age group (sourced from IHME’s GHDx database). In addition to premature deaths, we calculate the morbidity incidences related to smoke inhalation. On average, for each year between 2013 and 2017, our analysis has identified an additional 4390 respiratory hospital admissions, 635 thousand severe asthma symptom days among asthmatic children, and 8.9 million workdays lost in the working population.

## Discussion

### Uncertainties

Our study is the most comprehensive analysis of the long-term health effects of peatland fires in Indonesia carried out to date, in terms of the spatial and temporal cover and the application of CRFs to analyse the adult long-term mortality, infant deaths, and the additional morbidity effects associated with smoke inhalation in the exposed population. This improved knowledge leads to a better understanding of the negative health effects linked to peat drainage. Nevertheless, the analysis is subject to several important sources of uncertainty, related to both data and the models that we use. In terms of data, the main uncertainties pertain to: (i) the areas covered by peatlands; (ii) the occurrence of fires; and (iii) the estimation of PM_2.5_. In the last 20 years, 5 studies have published estimates of peatland occurrence in Sumatra and Kalimantan (for an overview, see [46]). For Sumatra, these estimates range from 5.6 million ha [47], to 9.6 million ha [48]. For Kalimantan, estimates range from 4.8 million ha [14] to 6.7 million ha [47]. In this study, the numbers of [14], are used, in line with the numbers formally adopted by the Government of Indonesia. Based on these numbers, the contribution of peatland fires to overall PM_2.5_ emitted from fires is estimated at 86% in Sumatra (average over 2013–217, range: 75% in 2017 to 94% in 2013) and 76% in Kalimantan (range: 62% in 2016 to 88% in 2013). This result is comparable to Kiely et al. [49], who estimate that, across Indonesia, peatland fires contributed 71% of total PM_2.5_ emissions from fires in Indonesia during September–October 2015. Our study focusses on two islands with extensive peat areas, which can explain why we find somewhat higher values. However, the uncertainty in peat area means for our study that there is uncertainty in the attribution of PM_2.5_ to peatland fires (as opposed to fires in mineral soil). Since the numbers on peat extent that we use are relatively low [14], we may underestimate the contribution of peatland fires to overall increases in PM_2.5_, and thereby underestimate the associated health effects.

Next, there is uncertainty regarding the occurrence of fires as measured with MODIS. The MODIS MCD64A1 product presents an estimate of the area burned in a given time period, however it is likely to underreport the actual burned area. In particular, small, burned areas (less than 100 ha) and burned areas in croplands and degraded grasslands are not all registered [16]. The estimates of burned areas presented in this report are subject to these same uncertainties. As for the measurement of PM_2.5_, we are combining ground observations of PM_2.5_ from the Government of Indonesia with models of ambient PM_2.5_ based on remote sensing observations. As explained in Section 3.2, our model has a good fit with the observed data, having an R^2^ of 0.90 with PM_2.5_ measurement stations and an R^2^ = 0.77 for all sites. However, we apply a simplification in the allocation of PM_2.5_ emissions to peatland fire versus forest fires on mineral land. In particular, we do not consider SOA formation in distinguishing the ratio PM_2.5_ from peatland fires versus forest fires. The incomplete burning of peat soils means that SOA formation may, on a per hectare basis, be higher in peatlands compared to forests on mineral land (e.g. [49]). Furtheromore, the EF we use for peat combustion (9.04 g/kg) is conservative. Other studies which have done field measurements have found up to 29.6 g/kg [50] and 22.3 g/kg [51]. Both aspects mean that we may underestimate the amount of ambient PM_2.5_ resulting from peatland fires (and overestimate the PM_2.5_ resulting from forest fires on mineral land). This means we may therefore underestimate the health effects of peatland fires.

In terms of health impact models, a key source of uncertainty lies in the selection of the concentration response functions that we use to assess health effects, and in our underlying input data assumptions. A first important assumption that we make is that the exposure to PM_2.5_, as measured over the period 2013–2017, is representative for the long-term exposure in Sumatra and Kalimantan. The period we study has two dry years (2014, 2015) two wet years (2016, 2017) and a year with average rainfall (2013). This can be considered representative for long-term rainfall conditions. Another key assumption is that PM_2.5_ emissions from other sources, in particular transportation, industry, energy supply and households, are similar in magnitude during the wet and dry season, so that the total increase in the dry season can be attributed to fires. There is no reason to assume any of these sources emits significantly more PM_2.5_ in the dry season compared to the wet season, however we cannot exclude that this may be the case. Furthermore, it may be that different rainfall patterns in the dry and wet season influence the rate at which PM_2.5_ is removed from the atmosphere affecting our assumption that the difference between the dry and the wet season PM_2.5_ concentrations can be solely attributed to biomass burning. The PM_2.5_ data for the year 2016, the year with the fewest peatland fires, show that the associated error is likely to be small (see Fig. 3). For 2016, the average Jan to May = 16.75 μg/m^3^, average Jan to Jul = 16.36 μg/m^3^, and average Jan to Dec = 16.66 μg/m^3^ – in other words, the year with the least peatland fires experienced fairly constant average PM_2.5_ concentrations throughout the year. An additional, important assumption is that a peak in PM_2.5_ concentration during the dry season can be averaged out over the year in order to assess health impacts; in other words that the health effects of a seasonal peak in PM_2.5_ are similar to those of a (lower) annual average increase in PM_2.5_. Since we are analyzing the long-term impacts of PM_2.5_ exposure, we believe the resulting potential errror to be small, and we note that this assumption is also made in various other studies analyzing the health impacts of forest fires, e.g. [42, 52]. Finally, we assume that the human toxicity of PM_2.5_ derived from peatland fires is in line with the average toxicity of PM_2.5_ (as related to health effects in concentration-response curves world-wide). However, various studies indicate that PM_2.5_ from biomass burning is relatively toxic, compared to other sources of PM_2.5_ such as road dust or aerosols from sea salt spray [53, 54].

Furthermore, there is uncertainty related to applying concentration-response curves to Indonesia, in particular because many of the underlying data are from Western Europe and the US, even though the study that we used for our base case estimate, [30], also includes data from China. This is reflected in the spread of the values that we present in Table 4. The accuracy of the application depends on the similarity of factors such as: (a) the chemical composition of the PM_2.5_; (b) the activity patterns and time spent outdoors; (c) underlying population disease profile and health status, including availability and utilization of health care services; (d) socioeconomic status; (e) age distribution, and (f) exposure to other pollutants, both outdoors and indoors. Of particular relevance is that Indonesia’s population is much younger and hospital admissions are much lower compared to the US and Western Europe. In addition, Indonesia and many other low- and middle-income countries have a different proportion of communicable diseases (around 16% of natural deaths) than the U.S. and Western Europe (around 5–6%). To explore the implications of using different CRFs, Table 4 below presents a sensitivity analysis, using three different choices for the CRF, with further details provided in Table A1.5 in the SM.

**Table 4:** Sensitivity analysis, adult premature deaths (mean air concentrations 2013–17)

Location	Vodonos (low/high = 95%CI)			HRAPIE	Crouse	Burnett
	Central	Low	High	Central	Central	Central
Sumatra	23,850	22,450	25,730	14,310	46,720	13,810
Kalimantan	9270	8730	10,000	5570	18,110	6050
Total	33,130	31,180	35,720	19,870	64,830	19,870

Numbers may not add up due to rounding

Finally, we need to stress that a limitation of our analysis is that we only examine premature mortality and morbidity effects of peatlands fires in Sumatra and Kalimantan. In years with severe fires, such as 2014 and 2015, smoke from peatland fires is spread over considerably larger areas, including other islands of Indonesia, Malaysia, Singapore and even parts of Thailand. Even though the health effects are concentrated in the two islands that we examine, mortality and morbidity effects do occur in other areas, both as a consequence of peatland fires in Sumatra and Kalimantan and due to peatland fires in other areas (e.g. Papua and Sulawesi) [55–57]. Consequently, our numbers are an underestimate of the overall health effects of peatland fires in Indonesia.

Our results can be compared to two other studies that assessed the health effects of peatland fires in Sumatra and Kalimantan. Crippa et al. [56] find that short-term exposure to air pollution resulting from peat fires in 2015 may have caused 11,880 (6153–17,270) excess mortalities across SE Asia and state that “the estimated deaths represent only a fraction of the overall premature fatalities due to long-term exposure to unhealthy air quality conditions”. They estimate that ~ 75,600 excess premature mortality would occur each year if the population received long-term exposure to the pollutant concentrations experienced in fall 2015 [56]. Kiely et al. (2020) [12] analysed excess mortality from health effects related to peat fires in six dry years with extensive peat fires in the period 2004 to 2015. For 2015, they found that exposure to PM resulted in 44,040 excess deaths in SE Asia, of which 61% (about 27,000 deaths) occurred in Sumatra and Kalimantan [12]. Our estimate for 2015 is 45,300 premature deaths across Sumatra and Kalimantan due to peatland fires, a value that is halfway between the estimate of the two studies [12, 55]. A main factor driving the differences between the studies is the counterfactual, i.e. the amount of PM_2.5_ that is added to the background PM_2.5_ concentration due to peatland fires (for instance, [12] assumed this to be 25 μg/m^3^, whereas our counterfactual is 18 μg/m^3^), but also differences in input data such as share of total PM_2.5_ emissions from peatland compared to other fires, population exposure-level, and demographic data play a role.

### Policy implications

The use and management of Indonesian peatlands is challenging. Peatlands cover large areas (between 15 and 20 million ha), often located in remote areas, in particular in Sumatra, Kalimantan and Papua. These peatlands are an important economic asset, given that they can be used for agriculture [47, 58, 59]. At present, only small areas of peatlands are being used for crops that do not require drainage, such as sago. The drainage of peatlands for other crops brings significant negative externalities. In addition to adverse health effects from peatland fires, these include CO_2_ emissions, loss of biodiversity, and soil subsidence that will, over time, increase flood risks across Indonesian lowlands [60–62]. Importantly, fire occurrence in 2016 and 2017 was markedly lower, which may be related to higher rainfall in 2016 and 2017, compared to 2014 and 2015. Fire occurrence increased again in 2019 [63].

We show that the health effects of draining and converting peatlands for agricultural development are substantial. Annual premature adult mortality due to peatland fires ranges from 19,900 to 64,800 cases, depending upon the concentration-response curve used (Table 4). Clearly, also the lower end of the model results indicates a substantial health effect attributable to peatland fires (6% of adult natural mortality). Furthermore, peatland fires put additional stress on the country’s health care system, and result in lost workdays due to air pollution related sicknesses. Both aspects are economic costs for Indonesia. There have also been reports that reduced lung functioning due to air pollution increases population vulnerability to COVID-19 [64]. The relation between COVID-19 and air pollution from peat and forest fires remains to be further studied, but this may provide further policy incentive to address peatland fires.

The Indonesian government has already banned farmers from dry-season crop residue burning, and such efforts are very important in mitigating health risks. In addition, health risks should be considered in land use planning and decision making on peatland uses. It is relevant to consider in which land use types the fires predominantly take place. Our data show that the least fire-prone areas are forests, which under natural conditions burn very seldomly (0.1% of the forest land burns each year, an average over the period 2013–2017, in Sumatra and Kalimantan; part of this land is burned deliberately to clear space for agriculture). On the other end of the spectrum, each year some 4% of agricultural lands and some 6% of grasslands burn. Forest plantations and perennial (plantation) crops take an intermediate position; some 2% is burned each year on average across Sumatra and Kalimantan. Around 61% of PM_2.5_ emissions related to biomass are from grasslands and shrublands, and 23% from plantations (annual croplands cover a much smaller area and contribute only 7%).

To reduce fire risks and associated health effects, a first priority is the rehabilitation of degraded grasslands and shrublands in peat, involving rewetting, revegetating, and controlling fire, cf. [65]. Second, in the near to medium term, there is a need to phase out oil palm, acacia, coconut and other crops on peat that require drainage, and replace these by other peatland uses that do not require drainage. It is noted that low water tables in plantations extend to adjacent areas [66]; drainage for plantations desiccates adjacent areas up to several km from the plantation boundary. Hence, drainage in plantations also increases fire risks in other land use types in peat including in forests and grasslands.

In the future, fire risks from plantation agriculture in peat involving drainage will only increase. Drainage leads to soil subsidence. As shown in [67], most drained plantations in peat have an expected lifecycle of 1 to 3 rotations (25 to 75 years) before they are subsided to the level that seasonal flooding kills the trees (both oil palm and acacia are sensitive to high water tables). Subsequently, the plantation will be abandoned. Abandoned plantations have a very high fire risk: their drained status and ample dead biomass mean fires will propagate quickly in the dry season. When the area covered by abandoned plantations increases in the coming decades, fire risks will further increase. This again points to the need to develop new plantation models in peat, using paludiculture (no drainage) crops such as sago [46]. This should be done as soon as possible, before ongoing peat subsidence and increasing flood and fire risks constrain the possibilities for paludiculture, since most paludiculture crops such as sago and jelutong are also sensitive to flooding and fire.

## Conclusions

Indonesian peatlands have been drained for agricultural development for several decades. Although this has contributed to economic development through large-scale and, to a lesser degree, smallholder plantations, peat drainage is increasingly causing environmental externalities with adverse economic impacts. One of these is air pollution resulting from peatland fires. Peatland fires occur every year, even though their extent is much larger in dry (El Niño) years. Peatland fires, therefore, increase the long-term exposure of people to air pollution. We find that PM_2.5_ air pollution from peatland fires causes, on average, around 33,100 adults and 2900 infants to die prematurely from air pollution, in the islands of Sumatra and Kalimantan alone. In addition, peatland fires cause around 4390 additional hospital admissions related to respiratory diseases, 635 thousand cases of severe asthma attacks in children, and 8.9 million lost work-days each year in these two islands. Sumatra and Kalimantan have the country’s highest concentrations of smoke from peatland fires, but there will also be health impacts of peatland fires in other Indonesian islands and in neighboring countries, in particular Singapore and Malaysia [68]. The main source of uncertainty is in the CRF that we use, with different CRFs leading to annual premature adult mortality ranging from 19,900 to 64,800 cases. Currently, the population of both islands is relatively young. With aging of the population over time, vulnerabilities to air pollution and health effects from peatland fires will increase. Hence, it is critical that health effects from peatland fires are acknowledged and considered in decision making, providing an additional element in support of current Government of Indonesia policies to reduce drainage-based agriculture in peatlands.

## Supplementary Information

**Additional file 1: **A1.1 Occurrence of Peatland fires. **Table A1.** Burned peat and mineral lands by land cover class, 2013 to 2017 (hectare). A1.2 Analysis of monthly PM2.5 concentrations. PM_2.5_ concentrations are estimated using satellite Aerosol Optical Depth (AOD). AOD is a measure of the extinction (scattering and absorption) of light by aerosols in a column of air from the Earth’s surface up to space. The AOD is used to represent the abundance of total aerosol particles in an atmospheric column [17]. To analyze AOD, we use data from three satellite instruments: twin MODIS (MODerate resolution Imaging Spectroradiometer) instruments and the MISR (Multi-angle Imaging Spectroradiometer) instrument [69]. We retrieve AOD with two algorithms that process MODIS radiances on both the Terra and Aqua satellites: Dark Target (DT) and Deep Blue (DB). The DT retrieval algorithm [70], is designed to retrieve AOD over dark surfaces (e.g. vegetated land surfaces and dark soils). The DB retrieval algorithm [71], uses blue wavelength measurements where the surface reflectance over land is typically much lower than at longer wavelengths, allowing for the retrieval of aerosol properties over both bright and dark surfaces. This study uses the recently released collection 6.1 of the MODIS retrieved AOD products, which include spatial resolution of 10 km and several updates to the DT [72], and DB algorithms [71, 73]. The MISR instrument [74], retrieval algorithm uses the same-scene multi-angular views provided by the nine view- angles to solve for surface and top-of-atmosphere reflectance contributions, providing AOD retrievals over bright and dark land surfaces without absolute surface reflectance assumptions [75]. Specifically, we use AOD retrieved from the recently released MISRv23 algorithm [76], which provides AOD at a spatial resolution of 4.4 km. Data from MODIS and MSIR is resampled at 4.4 km resolution and combined based on their relative uncertainties with Aerosol Robotic Network (AERONET) AOD ground measurements [17, 77, 78]. AERONET is a global sun photometer network established by NASA and PHOTONS (PHOtométrie pour le Traitement Opérationnel de Normalisation Satellitaire) [79]. This study uses AOD at 550 nm from level 2 of the version 3 AERONET data [80]. To convert the combined AOD to surface PM_2.5_ concentrations, we use the simulated ratio of total column AOD to surface PM_2.5_. The ratio of total column AOD to surface PM_2.5_ is a function of the factors that relate PM_2.5_ mass to satellite observations of AOD (e.g., aerosol size, aerosol composition, diurnal variation, relative humidity, and the vertical structure of aerosol extinction [81]. We simulate this relationship using the GEOS-Chem chemical transport model. A full description of the GEOS-Chem simulation used can be found in [17]. We use v11–01 of GEOS-Chem, and our simulation is driven by assimilated meteorological data from the MERRA-2 Reanalysis of the NASA Global Modeling and Assimilation Office (GMAO) [82]. The simulation is conducted for the years 2013 to 2017 with 47 vertical layers at a spatial resolution of 0.5° × 0.625° (~ 50 km × 60 km) across all of Asia (including Indonesia). The top of lowest model layer is ~ 100 m. Anthropogenic emissions of aerosols and their precursors are provided by the MIX inventory [83]. Biomass burning emissions are provided for individual years by the GFED4 open fire emissions inventory. A1.3 Emission factors. **Table A2.** Emission factors for above ground biomass (kg PM_2.5_ per kg dry biomass). **Table A3.** Above ground biomass per hectare. Table A4. Emissions from peat biomass burning. **Table A5.** Emissions from biomass burning, per hectare. A1.4. Concentration-Response functions. The meta-analysis study included 53 cohort studies, 39 studies from North America, eight from Europe, and six from Asia [30]. The mean concentration of PM_2.5_ across the studies was 15.7 μg/m^3^, with higher concentrations observed in the Asian studies with a mean of 30.5 μg/m^3^. The authors’ analysis indicated robust association between PM_2.5_ and premature mortality. At the mean concentration of 15.7 μg/m^3^, a 10 μg/m^3^ increase in PM_2.5_ was associated with a 10.3% (95% confidence interval (CI) of 9.7 to 11.1%) increase in adult premature mortality. This risk function was used for the base case estimates since it included the greatest number of studies in total, with a few from Asia. To examine the sensitivity of the results to the choice of this study, adult mortality estimates were generated from three other cohort studies (Section 4.3). The health risk was applied to the mortality rate in the population over 30 years old (Section 4.1.3) [30]. A second estimate of adult mortality by Burnett et al. (2018) [9] involved a meta-analysis of 41 cohort studies from around the world while allowing for flexibility in determining the shape of the CRF. The derived CRF was an improvement over previous risk functions used for the GBD estimates, since it added studies from countries with very high concentrations of PM_2.5_ such as China, and relied solely on air pollution studies (previous CRF for GBD incorporated other combustion sources of PM_2.5_ such as secondhand smoke and household air pollution). In addition, the CRF is restricted to mortality from non-communicable diseases (NCD) and lower respiratory illness (LRI), rather than all-causes, since these two outcomes have been closely linked to PM_2.5_ exposure in both mortality and morbidity studies. The resulting CRF is non-linear and fairly complex statistically. The Burnett et al. (2018) risk function, [9], was applied to the NCD + LRI mortality rate in the population older than 25 years (Section 4.1.3). Figure A2.1 shows the shape of the Burnett et al. (2018) function, along with the other studies used for the CRF. A third analysis was based on Crouse et al. (2020) [37], who utilized census data on 2.4 million Canadian adults to examine non-accidental and cause-specific mortality between 2001 and 2011. PM_2.5_ exposures were assigned to participants’ residence using satellite-based estimates. Of particular interest, the study compared impacts of using three different average exposure periods (1, 3, and 8 years), so it is relevant to the five-year average of concentrations due to peatland fires. For a 10 μg/m^3^ change in PM_2.5_ the associated with risk estimates for non-accidental mortality of approximately 11% (95% CI = 8, 13%), 20% (95% CI = 17, 23%) and 23% (95% CI = 20, 27%), respectively, for one-, three- and eight-years of average prior exposure. Existing studies indicate the effects of exposure changes can be experienced within one or 2 years [84–86], but [37], found larger risks when even longer exposure times were considered. For the sensitivity analysis, the risk associated with 3 years of exposure was used to approximate the impact of the five-years of exposure from the peatland fires, and the risk associated with 1 year of exposure was used for calculating the impact of the single year (2015). The Crouse et al. (2020) health risk [37], was applied to the mortality rate in the population over 30 years old. The fourth, and final, study used in the sensitivity analysis is based on the recommendations from WHO’s HRAPIE (2013) review [7], which was based primarily on an earlier meta-analysis [8]. The estimated risk for all-cause mortality (for the population 30+) was based on eleven studies available at the time. The resulting risk was 6% (95% CI = 4, 8%). As shown in Fig. 6, the CRF from Vodonos et al. (2018) [30] lies in between those of the three other studies. Fig. A1. Approximate change in mortality risk vs PM_2.5_ concentration for different published studies. The risk estimates from two recent cohort studies conducted in China are presented for comparison with the studies used for the base case and sensitivity analysis. Li et al. (2018) examined a cohort of over 13,000 participants from the Chinese Longitudinal Healthy Longevity Survey [87]. PM_2.5_ exposures were assigned to residential locations and derived from remote sensing satellite data at a 1 km grid resolution. The median concentration was 50.7 μg/m^3^. The estimated all-cause mortality risk for a 10 μg/m^3^ change in PM_2.5_ was 8% (95% CI = 6, 9%). Another study [88] (which was included in the meta-analyses of Vodonos and Burnett cited above) analysed a cohort of 190,000 men randomly selected from 45 of China’s 145 Disease Surveillance Points around the country. PM_2.5_ concentrations were developed by combining data from remote sensing satellites, chemical transport model and ground-based monitors. With a mean PM_2.5_ concentration of 43.7 μg/m^3^, the non-accidental mortality risk estimate for a 10 μg/m^3^ change in PM_2.5_ was 9% (95% CI = 8, 9%). A1.5. Sensitivity analysis. **Table A6.** Sensitivity analysis of alternative assumptions for adult and infant mortality.

## Data Availability

The datasets during and/or analysed during the current study available from the corresponding author on reasonable request.

## Footnotes

1 https://sedac.ciesin.columbia.edu/data/set/gpw-v4-population-count-rev11

2 Information is available online: http://ghdx.healthdata.org/gbd-results-tool
