# Supplementary material for: The health impacts of Indonesian peatland fires
Source: Environ Health. 2022 Jul 6;21:62. doi: 10.1186/s12940-022-00872-w (PMC9256533; doi:10.1186/s12940-022-00872-w)
Supplement: Supplementary file 1 — Additional file 1: A1.1 Occurrence of Peatland fires. Table A1. Burned peat and mineral lands by land cover class, 2013 to 2017 (hectare). A1.2 Analysis of monthly PM2.5 concentrations. PM2.5 concentrations are estimated using satellite Aerosol Optical Depth (AOD). AOD is a measure of the extinction (scattering and absorption) of light by aerosols in a column of air from the Earth’s surface up to space. The AOD is used to represent the abundance of total aerosol particles in an atmospheric column [17]. To analyze AOD, we use data from three satellite instruments: twin MODIS (MODerate resolution Imaging Spectroradiometer) instruments and the MISR (Multi-angle Imaging Spectroradiometer) instrument [69]. We retrieve AOD with two algorithms that process MODIS radiances on both the Terra and Aqua satellites: Dark Target (DT) and Deep Blue (DB). The DT retrieval algorithm [70], is designed to retrieve AOD over dark surfaces (e.g. vegetated land surfaces and dark soils). The DB retrieval algorithm [71], uses blue wavelength measurements where the surface reflectance over land is typically much lower than at longer wavelengths, allowing for the retrieval of aerosol properties over both bright and dark surfaces. This study uses the recently released collection 6.1 of the MODIS retrieved AOD products, which include spatial resolution of 10 km and several updates to the DT [72], and DB algorithms [71, 73]. The MISR instrument [74], retrieval algorithm uses the same-scene multi-angular views provided by the nine view- angles to solve for surface and top-of-atmosphere reflectance contributions, providing AOD retrievals over bright and dark land surfaces without absolute surface reflectance assumptions [75]. Specifically, we use AOD retrieved from the recently released MISRv23 algorithm [76], which provides AOD at a spatial resolution of 4.4 km. Data from MODIS and MSIR is resampled at 4.4 km resolution and combined based on their relative uncertainties with Aerosol Robotic Netwo [file 12940_2022_872_MOESM1_ESM.docx]

# Supplementary Materials

A1.1 Occurrence of Peatland fires

Table A1. Burned peat and mineral lands by land cover class, 2013 to 2017 (hectare).

| *Sumatra* | **2013** | **2014** | **2015** | **2016** | **2017** | **Average** |
| --- | --- | --- | --- | --- | --- | --- |
| **Mineral soil** |  |  |  |  |  |  |
| Forests (including degraded forests) | 5731 | 28994 | 15881 | 1773 | 1200 | 10716 |
| Plantation and forest plantation | 18081 | 43792 | 26771 | 14710 | 6033 | 21878 |
| Degraded lands (bare ground, wet/dry shrubland and savanna and grass) | 68075 | 190573 | 364364 | 29804 | 8581 | 132279 |
| Agricultural lands | 6916 | 38658 | 46737 | 1629 | 625 | 18913 |
| **Peat soils** |  |  |  |  |  |  |
| Forests (including degraded forests) | 10188 | 32695 | 6176 | 1773 | 467 | 10260 |
| Plantation and forest plantation | 32144 | 49383 | 10411 | 14710 | 2346 | 21799 |
| Degraded lands (bare ground, wet/dry shrubland and savanna and grass) | 121022 | 214901 | 141697 | 29804 | 3337 | 102152 |
| Agricultural lands | 12295 | 43593 | 18175 | 1629 | 243 | 15187 |
| **Total** | **274452** | **642589** | **630214** | **95833** | **22831** | **333184** |
| *Kalimantan* |  |  |  |  |  |  |
| **Mineral soil** |  |  |  |  |  |  |
| Forests (including degraded forests) | 3360 | 57637 | 26820 | 1425 | 1453 | 18139 |
| Plantation and forest plantation | 10600 | 87056 | 45212 | 11821 | 7303 | 32399 |
| Degraded lands (bare ground, wet/dry shrubland and savanna and grass) | 39911 | 378846 | 615339 | 23950 | 10388 | 213687 |
| Agricultural lands | 4055 | 76850 | 78929 | 1309 | 756 | 32380 |
| **Peat soils** |  |  |  |  |  |  |
| Forests (including degraded forests) | 2749 | 36850 | 8470 | 271 | 386 | 9745 |
| Plantation and forest plantation | 8673 | 55659 | 14277 | 2252 | 1941 | 16560 |
| Degraded lands (bare ground, wet/dry shrubland and savanna and grass) | 32654 | 242213 | 194317 | 4562 | 2761 | 95302 |
| Agricultural lands | 3318 | 49134 | 24925 | 249 | 201 | 15565 |
| **Total** | **105319** | **984245** | **1008289** | **45839** | **25189** | **433776** |

**A1.2 Analysis of monthly PM_2.5_ concentrations**

PM_2.5_ concentrations are estimated using satellite Aerosol Optical Depth (AOD). AOD is a measure of the extinction (scattering and absorption) of light by aerosols in a column of air from the Earth’s surface up to space. The AOD is used to represent the abundance of total aerosol particles in an atmospheric column [17]. To analyze AOD, we use data from three satellite instruments: twin MODIS (MODerate resolution Imaging Spectroradiometer) instruments and the MISR (Multi-angle Imaging Spectroradiometer) instrument [69]. We retrieve AOD with two algorithms that process MODIS radiances on both the Terra and Aqua satellites: Dark Target (DT) and Deep Blue (DB). The DT retrieval algorithm [70], is designed to retrieve AOD over dark surfaces (e.g. vegetated land surfaces and dark soils). The DB retrieval algorithm [71], uses blue wavelength measurements where the surface reflectance over land is typically much lower than at longer wavelengths, allowing for the retrieval of aerosol properties over both bright and dark surfaces. This study uses the recently released collection 6.1 of the MODIS retrieved AOD products, which include spatial resolution of 10 km and several updates to the DT [72], and DB algorithms [71,73]. The MISR instrument [74], retrieval algorithm uses the same-scene multi-angular views provided by the nine view- angles to solve for surface and top-of-atmosphere reflectance contributions, providing AOD retrievals over bright and dark land surfaces without absolute surface reflectance assumptions [75]. Specifically, we use AOD retrieved from the recently released MISRv23 algorithm [76], which provides AOD at a spatial resolution of 4.4 km. Data from MODIS and MSIR is resampled at 4.4 km resolution and combined based on their relative uncertainties with Aerosol Robotic Network (AERONET) AOD ground measurements [17,77, 78]. AERONET is a global sun photometer network established by NASA and PHOTONS (PHOtométrie pour le Traitement Opérationnel de Normalisation Satellitaire) [79]. This study uses AOD at 550 nm from level 2 of the version 3 AERONET data [80].

To convert the combined AOD to surface PM_2.5_ concentrations, we use the simulated ratio of total column AOD to surface PM_2.5_. The ratio of total column AOD to surface PM_2.5_ is a function of the factors that relate PM_2.5_ mass to satellite observations of AOD (e.g., aerosol size, aerosol composition, diurnal variation, relative humidity, and the vertical structure of aerosol extinction [81] . We simulate this relationship using the GEOS-Chem chemical transport model. A full description of the GEOS-Chem simulation used can be found in [17]. We use v11-01 of GEOS-Chem, and our simulation is driven by assimilated meteorological data from the MERRA-2 Reanalysis of the NASA Global Modeling and Assimilation Office (GMAO) [82]. The simulation is conducted for the years 2013 to 2017 with 47 vertical layers at a spatial resolution of 0.5° x 0.625° (~ 50 km x 60 km) across all of Asia (including Indonesia). The top of lowest model layer is ~100 m. Anthropogenic emissions of aerosols and their precursors are provided by the MIX inventory [83]. Biomass burning emissions are provided for individual years by the GFED4 open fire emissions inventory

**A1.3 Emission factors**

Table A2. Emission factors for above ground biomass (kg PM_2.5_ per kg dry biomass)

| Land Cover Class | Emission factor |
| --- | --- |
| Forests (including degraded forests) | 9 |
| Plantation and forest plantation ^/1^ | 9 |
| Degraded lands (bare ground. wet/dry shrub. savanna and grass) ^/1^ | 6.6 |
| Agricultural lands (rice straw) ^/2^ | 4.2 |

Sources: /1 [27] /2 [28]

Table A3. Above ground biomass per hectare

| Land Cover Class | Avergae above ground biomass, dry weight (ton/ha) |
| --- | --- |
| Forests (including degraded forests) ^/1^ | 390 |
| Plantation and forest plantation ^/1, 2^ | 133 |
| Degraded lands ^/1^ | 30 |
| Agricultural lands ^/3^ | 7 |

| Sources: /1 [84] ; /2 [85] /3 [86] |
| --- |

Table A4. Emissions from peat biomass burning

| Peat biomass burned per hectare: 504.9 tonnes/ha ^/1,2, 3^ |
| --- |
| Emission of PM_2.5_ per unit biomass: 9.04 g PM_2.5_/kg biomass^/4^ |

Sources: 1/[23]; 2/[24]; 3/[25]; 4/[26]

Table A5. Emissions from biomass burning, per hectare

| *Sumatra* | **2013** | **2014** | **2015** | **2016** | **2017** | **Average** |
| --- | --- | --- | --- | --- | --- | --- |
| **Mineral soil** |  |  |  |  |  |  |
| Forests (including degraded forests) | 2% | 5% | 5% | 2% | 9% | 5% |
| Plantation and forest plantation | 2% | 3% | 3% | 6% | 15% | 6% |
| Degraded lands (bare ground, wet/dry shrubland and savanna and grass) | 1% | 2% | 7% | 2% | 4% | 3% |
| Agricultural lands | 0% | 0% | 0% | 0% | 0% | 0% |
| **Peat soils** |  |  |  |  |  |  |
| Forests (including degraded forests) | 9% | 13% | 5% | 5% | 8% | 8% |
| Plantation and forest plantation | 19% | 14% | 6% | 30% | 28% | 20% |
| Degraded lands (bare ground, wet/dry shrubland and savanna and grass) | 60% | 52% | 66% | 51% | 33% | 52% |
| Agricultural lands | 6% | 10% | 8% | 3% | 2% | 6% |
| **Total** | 100% | 100% | 100% | 100% | 100% | 100% |
| **Total Peat** | **94%** | **90%** | **84%** | **89%** | **72%** | **86%** |
| *Kalimantan* | **2013** | **2014** | **2015** | **2016** | **2017** | **Average** |
| **Mineral soil** |  |  |  |  |  |  |
| Forests (including degraded forests) | 4% | 8% | 6% | 8% | 12% | 8% |
| Plantation and forest plantation | 5% | 4% | 4% | 23% | 20% | 11% |
| Degraded lands (bare ground, wet/dry shrubland and savanna and grass) | 3% | 3% | 8% | 8% | 5% | 5% |
| Agricultural lands | 0% | 0% | 0% | 0% | 0% | 0% |
| **Peat soils** |  |  |  |  |  |  |
| Forests (including degraded forests) | 8% | 12% | 5% | 4% | 7% | 7% |
| Plantation and forest plantation | 18% | 13% | 6% | 21% | 25% | 17% |
| Degraded lands (bare ground, wet/dry shrubland and savanna and grass) | 56% | 48% | 63% | 35% | 30% | 47% |
| Agricultural lands | 6% | 9% | 8% | 2% | 2% | 5% |
| **Total** | **100%** | **100%** | **100%** | **100%** | **100%** | **100%** |
| **Total peat** | **88%** | **84%** | **81%** | **61%** | **64%** | **76%** |

**A1.4. Concentration-Response functions**

The meta-analysis study included 53 cohort studies, 39 studies from North America, eight from Europe, and six from Asia [30]. The mean concentration of PM_2.5_ across the studies was 15.7 μg/m^3^, with higher concentrations observed in the Asian studies with a mean of 30.5 μg/m^3^. The authors’ analysis indicated robust association between PM_2.5_ and premature mortality. At the mean concentration of 15.7 μg/m^3^, a 10 μg/m^3^ increase in PM_2.5_ was associated with a 10.3% (95% confidence interval (CI) of 9.7% to 11.1%) increase in adult premature mortality. This risk function was used for the base case estimates since it included the greatest number of studies in total, with a few from Asia. To examine the sensitivity of the results to the choice of this study, adult mortality estimates were generated from three other cohort studies (Section 4.3). The health risk was applied to the mortality rate in the population over 30 years old (Section 4.1.3) [30].

A second estimate of adult mortality by Burnett et al. (2018) [9] involved a meta-analysis of 41 cohort studies from around the world while allowing for flexibility in determining the shape of the CRF. The derived CRF was an improvement over previous risk functions used for the GBD estimates, since it added studies from countries with very high concentrations of PM_2.5_ such as China, and relied solely on air pollution studies (previous CRF for GBD incorporated other combustion sources of PM_2.5_ such as secondhand smoke and household air pollution). In addition, the CRF is restricted to mortality from non-communicable diseases (NCD) and lower respiratory illness (LRI), rather than all-causes, since these two outcomes have been closely linked to PM_2.5_ exposure in both mortality and morbidity studies. The resulting CRF is non-linear and fairly complex statistically. The Burnett et al. (2018) risk function, [9], was applied to the NCD+LRI mortality rate in the population older than 25 years (Section 4.1.3). Figure A2.1 shows the shape of the Burnett et al. (2018) function, along with the other studies used for the CRF.

A third analysis was based on Crouse et al. (2020) [37], who utilized census data on 2.4 million Canadian adults to examine non-accidental and cause-specific mortality between 2001 and 2011. PM_2.5_ exposures were assigned to participants’ residence using satellite-based estimates. Of particular interest, the study compared impacts of using three different average exposure periods (1, 3, and 8 years), so it is relevant to the five-year average of concentrations due to peatland fires. For a 10 μg/m^3^ change in PM_2.5_ the associated with risk estimates for non-accidental mortality of approximately 11% (95% CI = 8%, 13%), 20% (95% CI = 17%, 23%) and 23% (95% CI = 20%, 27%), respectively, for one-, three- and eight-years of average prior exposure. Existing studies indicate the effects of exposure changes can be experienced within one or two years [87,88,89], but [37], found larger risks when even longer exposure times were considered. For the sensitivity analysis, the risk associated with three years of exposure was used to approximate the impact of the five-years of exposure from the peatland fires, and the risk associated with one year of exposure was used for calculating the impact of the single year (2015). The Crouse et al. (2020) health risk [37], was applied to the mortality rate in the population over 30 years old.

The fourth, and final, study used in the sensitivity analysis is based on the recommendations from WHO’s HRAPIE (2013) review [7], which was based primarily on an earlier meta-analysis [8]. The estimated risk for all-cause mortality (for the population 30+) was based on eleven studies available at the time. The resulting risk was 6% (95% CI = 4%, 8%). As shown in Figure 6, the CRF from Vodonos et al. (2018) [30] lies in between those of the three other studies.


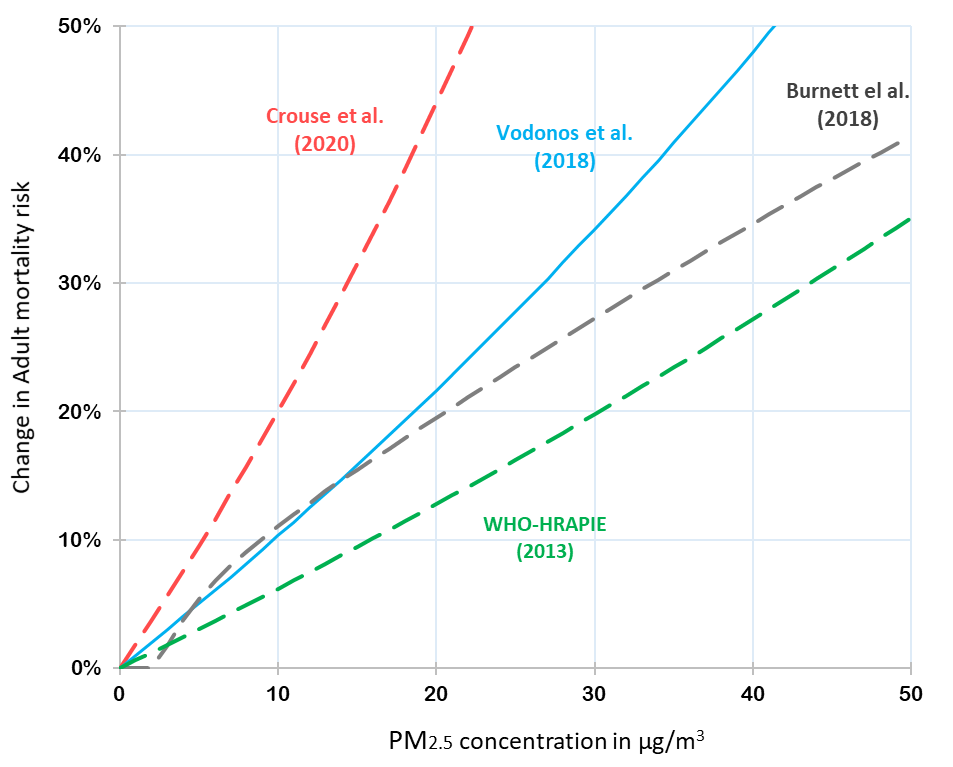


Figure A1. Approximate change in mortality risk vs PM_2.5_ concentration for different published studies.

The risk estimates from two recent cohort studies conducted in China are presented for comparison with the studies used for the base case and sensitivity analysis. Li et al. (2018) examined a cohort of over 13,000 participants from the Chinese Longitudinal Healthy Longevity Survey [90]. PM_2.5_ exposures were assigned to residential locations and derived from remote sensing satellite data at a one km grid resolution. The median concentration was 50.7 μg/m³. The estimated all-cause mortality risk for a 10 μg/m³ change in PM_2.5_ was 8% (95% CI = 6%, 9%). Another study [91] (which was included in the meta-analyses of Vodonos and Burnett cited above) analysed a cohort of 190,000 men randomly selected from 45 of China’s 145 Disease Surveillance Points around the country. PM_2.5_ concentrations were developed by combining data from remote sensing satellites, chemical transport model and ground-based monitors. With a mean PM_2.5_ concentration of 43.7 μg/m³, the non-accidental mortality risk estimate for a 10 μg/m³ change in PM_2.5_ was 9% (95% CI = 8%, 9%).

**A1.5. Sensitivity analysis**

Table A6. Sensitivity analysis of alternative assumptions for adult and infant mortality

|  | **PM_2.5_**  **Fire-related** | | **PM_2.5_**  **Counterfactual** | | **Mortality rate** | | **CRF**  **Used** | | **Estimated Annual**  **Mortality** | | |  |
| --- | --- | --- | --- | --- | --- | --- | --- | --- | --- | --- | --- | --- |
| **Adult Mortality** | | | | | | | | | | |  |  |
| **Base Case** | | 5-yr ave  June-Dec | | 5-yr ave  Jan-May | | GHE  [32] | | Vodonos | | 33,100 | | |
| **Sensitivity #1**  (change CRF) | | 5-yr ave  June-Dec | | 5-yr ave  Jan-May | | GHE  [32] | | Burnett Crouse  HRAPIE | | 19,900  64,800  19,900 | | |
| **Sensitivity #2**  (change counterfactual) | | 5-yr ave  June-Dec | | 5-yr ave  Jan | | GHE  [32] | | Vodonos | | 38,300 | | |
| **Sensitivity #3**  (change mortality rate) | | 5-yr ave  June-Dec | | 5-yr ave  Jan-May | | IHME province-specific | | Vodonos | | 27,100 | | |
| **Sensitivity #4**  (year 2015) | | 2015  June-Dec | | 2015  Jan | | GHE  [32] | | Vodonos | | 98,400 | | |
| **Infant Mortality** | | | | | | | | | | |  |  |
| **Base Case** | | Year-specific  June-Dec | | Year-specific  Jan-May | | UN IGME [33] | | Heft-Neal | | 2,900 | | |
| **Sensitivity #5**  (change counterfactual) | | Year-specific  June-Dec | | Year-specific  Jan | | UN IGME [33] | | Heft-Neal | | 3,300 | | |
| **Sensitivity #6**  (change mortality rate) | | Year-specific  June-Dec | | Year-specific  Jan-May | | IHME province-specific | | Heft-Neal | | 2,800 | | |
| **Sensitivity #7**  (year 2015) | | 2015  June-Dec | | 2015  Jan | | UN IGME [33] | | Heft-Neal | | 7,900 | | |
